# Efficient implementation of analytical gradients for periodic hybrid functional calculations within fitted numerical atomic orbitals from NAO2GTO

**DOI:** 10.3389/fchem.2023.1232425

**Published:** 2023-07-27

**Authors:** Xinming Qin, Honghui Shang, Jinlong Yang

**Affiliations:** ^1^ Hefei National Research Center for Physical Sciences at the Microscale, University of Science and Technology of China, Hefei, Anhui, China; ^2^ Key Laboratory of Precision and Intelligent Chemistry, University of Science and Technology of China, Hefei, Anhui, China; ^3^ Hefei National Laboratory, University of Science and Technology of China, Hefei, Anhui, China

**Keywords:** Hartree-Fock exchange, atomic forces, electron repulsion integral derivatives, NAO2GTO, fitted orbitals, integral screening, linear scaling

## Abstract

The NAO2GTO scheme provides an efficient way to evaluate the electron repulsion integrals (ERIs) over numerical atomic orbitals (NAOs) with auxiliary Gaussian-type orbitals (GTOs). However, the NAO2GTO fitting will significantly impact the accuracy and convergence of hybrid functional calculations. To address this issue, here we propose to use the fitted orbitals as a new numerical basis to properly handle the mismatch between NAOs and fitted GTOs. We present an efficient and linear-scaling implementation of analytical gradients of Hartree-Fock exchange (HFX) energy for periodic HSE06 calculations with fitted NAOs in the HONPAS package. In our implementation, the ERIs and their derivatives for HFX matrix and forces are evaluated analytically with the auxiliary GTOs, while other terms are calculated using numerically discretized GTOs. Several integral screening techniques are employed to reduce the number of required ERI derivatives. We benchmark the accuracy and efficiency of our implementation and demonstrate that our results of lattice constants, bulk moduli, and band gaps of several typical semiconductors are in good agreement with the experimental values. We also show that the calculation of HFX forces based on a master-worker dynamic parallel scheme has a very high efficiency and scales linearly with respect to system size. Finally, we study the geometry optimization and polaron formation due to an excess electron in rutile TiO_2_ by means of HSE06 calculations to further validate the applicability of our implementation.

## 1 Introduction

The Kohn-Sham density-functional theory (KS-DFT) (Hohenberg and Kohn, 1964; Kohn and Sham, 1965) has become the most popular method for predicting the structural and electronic properties of molecular and condensed-matter systems. The success of DFT is attributed to the fact that the local-density approximation (LDA) (Kohn and Sham, 1965) and semilocal generalized-gradient approximation (GGA) (Perdew, 1985; Perdew et al., 1996) for exchange-correlation energy functional can provide reasonable accuracy at a low computational cost. However, local or semilocal functionals severely underestimate band gaps of semiconductors due to their intrinsic self-interaction error (Mori-Sánchez et al., 2008). Dramatic improvements can be achieved by incorporating a certain fraction of non-local orbital-dependent Hartree-Fock exchange (HFX) into the local or semilocal exchange, producing so-called hybrid functionals (Stephens et al., 1994; Adamo and Barone, 1999; Ernzerhof and Scuseria, 1999; Heyd et al., 2003, 2006; Krukau et al., 2006). In particular, the Heyd–Scuseria–Ernzerhof (HSE) screened hybrid functional (HSE03 (Heyd et al., 2003) or HSE06 (Heyd et al., 2006; Krukau et al., 2006)) is the most successful one in solid-state physics, which employs only a short-range HFX to avoid the problematic effects of long-range one in solids (Janesko et al., 2009). It has been shown that HSE can yield improved results of structural, thermochemical, and electronic properties for both molecules and solids (Paier et al., 2006b,a; Marsman et al., 2008; Henderson et al., 2011). Nevertheless, the evaluation of exact exchange is significantly more expensive than the local or semilocal approximations, which formally has a quartic scaling $O\left(N^{4}\right)$ with system size *N* and hinders the wide applications of hybrid functionals. As a result, it is of great importance to develop and implement efficient and linear-scaling approaches for large-scale hybrid functional calculations.

The success of hybrid functionals has also prompted the development of efficient numerical techniques for reducing the computational cost and scaling of HFX calculations in the past two decades. Currently, hybrid functional calculations for periodic systems are available in a range of DFT packages with plane-wave (PW) (Marsman et al., 2008; Spencer and Alavi, 2008; Broqvist et al., 2009; Hu et al., 2017b), Gaussian-type orbital (GTO) (Heyd et al., 2003; Guidon et al., 2008; Lee et al., 2022), and numerical atomic orbital (NAO) (Shang et al., 2011; Levchenko et al., 2015; Qin et al., 2015; Lin et al., 2020) basis sets. For PW basis sets, a low-rank approximation called adaptively compressed exchange (ACE) (Lin, 2016; Hu et al., 2017a) operator has been proposed, resulting in significant acceleration of hybrid functional calculations. When combined with the interpolative separable density fitting (ISDF) algorithm (Lu and Ying, 2015; Hu et al., 2017b), the overall computational scaling can be further reduced to $O\left(N^{3}\right)$ . However, linear-scaling hybrid functional calculations within PWs cannot be achieved unless extended KS orbitals are converted to maximally localized Wannier functions (Wu et al., 2009; Ko et al., 2020). To enable linear-scaling hybrid functional calculations, one has to exploit the sparsity of HFX matrix represented with real-space localized basis functions. In this context, GTOs exhibit a natural advantage since they are analytical and decay exponentially in real space. Within GTOs, four-center electron repulsion integrals (ERIs) for constructing the HFX matrix can be evaluated analytically (Reine et al., 2012) and a number of linear-scaling approaches (Burant et al., 1996; Schwegler and Challacombe, 1996; Schwegler et al., 1997; Ochsenfeld et al., 1998) existed in the quantum chemistry community can be used as valuable references. Because of this, GTO-based electronic structure packages such as CP2K(Kühne et al., 2020), CRYSTAL (Dovesi et al., 2020), Q-Chem (Lee et al., 2022), and Pyscf (Sun et al., 2020) have made great progress in periodic HFX calculations.

In fact, current linear-scaling electronic structure packages, such as SIESTA (Soler et al., 2002), CONQUEST (Torralba et al., 2008), OPENMX (Ozaki and Kino, 2005), FHI-aims (Blum et al., 2009), HONPAS(Qin et al., 2015; 2020a) and ABACUS(Li et al., 2016), prefer to adopt NAO basis sets. Compared to exponentially decayed GTOs, NAOs are strictly localized in real space, which provides greater convenience for linear-scaling calculations. However, hybrid functional calculations with NAOs are more challenging since the numerical evaluation of ERIs is much more time-consuming. To reduce the computational cost, three possible routes can be taken. The first route is to expand the products of NAOs in terms of PWs (Chen et al., 2018; Lin et al., 2020), and the computational cost of HFX can be asymptotically quadratic due to the locality of NAOs. The second route is to introduce low-rank approximations, such as the resolution-of-the-identity (RI) approach (Ren et al., 2012; Levchenko et al., 2015; Lin et al., 2020, 2021) and the ISDF decomposition (Qin et al., 2020b,c), which can significantly reduce the computational cost by avoiding four-center integrals. Furthermore, linear-scaling HFX calculation can be implemented by using the localized RI (LRI) approximation (Levchenko et al., 2015; Lin et al., 2020). The third route is to fit the NAOs with a linear combination of several GTOs so that the ERIs can also be calculated analytically with fitted GTOs. We have previously proposed this scheme called NAO2GTO (Shang et al., 2011) to take full advantages of both NAOs and GTOs. In conjunction with several integral screening techniques, HFX calculations based on the NAO2GTO scheme can be very efficient and scale linearly (Shang et al., 2011; Qin et al., 2015). In practice, however, the NAOs cannot be fitted accurately with a small number (e.g., 3–6) of GTOs, which will seriously affects the accuracy and even the convergence of a hybrid functional calculation. We can improve the results by increasing the number of GTOs, but too many GTOs will significantly increase the computational cost of ERIs. To effectively utilize the NAO2GTO scheme for NAO-based hybrid functional calculations, it is crucial to address the mismatch between NAOs and fitted GTOs.

On the other hand, there have been few reports on analytical energy gradients (atomic forces) for periodic hybrid functional calculations with NAOs to date. Atomic forces are defined as analytical gradients of total energy to atomic positions, which are required for geometry optimization and *ab initio* molecular dynamics simulations. In the PW method, the two-electron HFX term has no contribution to atomic forces according to the Hellmann–Feynman theorem (Feynman, 1939) since the PW basis set is orthogonal and independent of the atomic positions. However, the situation becomes more complicated for NAOs, where the atomic forces also include Pulay corrections (Pulay, 1969) due to changes in the basis functions with respect to atomic positions. For the HFX forces, it is necessary to compute the first derivatives of ERIs in order to obtain analytical gradients of the HFX energy. The NAO2GTO scheme combined with integral screening also provides an efficient way to analytically evaluate the ERI derivatives over NAOs. Therefore, the implementation of HFX forces with NAOs is relatively straightforward.

In this work, we aim to extend the linear-scaling approach for the HFX force calculations of periodic systems based on the NAO2GTO scheme in the HONPAS package. In our approach, the original NAOs are replaced by fitted GTOs, so as to eliminate the errors introduced by the NAO2GTO fitting as much as possible. The ERI derivatives are analytically evaluated with the NAO2GTO scheme, and the computational cost is reduced by using integral screening techniques, enabling linear-scaling HFX force calculations. A master-worker dynamic parallel strategy is also adopted to achieve high parallel efficiency. We benchmark the accuracy and efficiency of our implementation by performing HSE06 calculations for periodic systems and apply it to investigate the small polaronic behavior of excess electrons in rutile TiO_2_. The rest of the paper is organized as follows. Section 2 reviews the theoretical framework. Section 3 provides a detailed description of our approach and implementation. Section 4 validates the performance of our implementation. A summary is given in Section 5.

## 2 Theory

### 2.1 Hybrid functional for periodic systems

Hybrid functionals currently used in the generalized KS framework contain a fraction of non-local, exact HFX term. In the PBE0 hybrid functional (also known as PBEh or PBE1PBE) (Adamo and Barone, 1999; Ernzerhof and Scuseria, 1999), the exchange-correlation energy is written as

$$E_{\text{xc}}^{\text{PBE0}}=\frac{1}{4}E_{x}^{\text{HF}}+\frac{3}{4}E_{x}^{\text{PBE}}+E_{c}^{\text{PBE}}$$ (1)

where 25% HFX $E_{x}^{\text{HF}}$ is mixed with 75% Perdew-Burke–Ernzerhof (PBE) exchange $E_{x}^{\text{PBE}}$ , and the electronic correlation is still represented by the part of the PBE correlation $E_{c}^{\text{PBE}}$ . The inclusion of the HFX in PBE0 reduces the self-interaction error of the density functional, resulting in a substantial improvement over the parent PBE. However, the full-range (FR) HFX is computationally very demanding and may be problematic in solids. To address this issue, Heyd, Scuseria, and Ernzerhof (Heyd et al., 2003; Heyd et al., 2006) proposed to replace the long-range part of HFX in the PBE0 by a corresponding PBE counterpart. Then, the resulting expression for the HSE exchange–correlation energy is given by

$$E_{\text{xc}}^{\text{HSE}}=\frac{1}{4}E_{x}^{\text{SR,HF}}\left(\omega \right)+\frac{3}{4}E_{x}^{\text{SR,PBE}}\left(\omega \right)+E_{x}^{\text{LR,PBE}}\left(\omega \right)+E_{c}^{\text{PBE}}$$ (2)

where $E_{x}^{\text{SR,HF}}$ and $E_{x}^{\text{LR,PBE}}\left(\omega \right)$ is the short-range (SR) HFX and long-range (LR) PBE exchange energies, and $E_{x}^{\text{SR,PBE}}\left(\omega \right)$ is the SR exchange energy. *ω* is an adjustable screening parameter that defines the range separation, and *ω* is set to 0.11 Bohr^−1^ for HSE06 (Heyd et al., 2006). Such a treatment not only improves the computational convenience but also avoids the problematic effects of LR HFX in metals and semiconductors with narrow band gaps.

For periodic systems, the HFX energy per unit cell can be written as

$$E_{x}^{\text{HF}}=-\frac{1}{2}\underset{\sigma =\left\{\alpha ,\beta \right\}}{\sum }\overset{\text{BZ}}{\underset{k,q}{\sum }}\times \overset{\text{occ}}{\underset{i,j}{\sum }}\int_{\Omega }\int \psi_{ik}^{\sigma *}\left(r\right)\psi_{jq}^{\sigma }\left(r\right)\hat{v}\left(r,r^{\prime }\right)\psi_{jq}^{\sigma *}\left(r^{\prime }\right)\psi_{ik}^{\sigma }\left(r^{\prime }\right)\;dr^{\prime }\;dr$$ (3)

where $\psi_{ik}^{\sigma }\left(r\right)$ denote the *i*-th occupied (occ) crystalline spin-orbitals with spin *σ* for **k** point sampling in the Brillouin zone (BZ), Ω is the unit cell volume. $E_{x}^{\text{HF}}$ is used to represent the FR or SR HFX energy, and $\hat{v}\left(r,r^{\prime }\right)$ is either the Coulomb operator $\hat{v}\left(r,r^{\prime }\right)=1/|r-r^{\prime }|$ in PBE0 or the screened Coulomb operator $\hat{v}\left(r,r^{\prime }\right)=erfc\left(\omega |r-r^{\prime }|\right)/|r-r^{\prime }|$ in HSE. Hereafter, we will formulate only the collinear spin-polarized case with *σ* = {*α*, *β*}.

In the linear combination of atomic orbitals (LCAO) method, the spin-orbitals are expanded in terms of a linear combination of Bloch basis functions

$$\psi_{ik}^{\sigma }\left(r\right)=\frac{1}{\sqrt{N}}\overset{N}{\underset{n}{\sum }}e^{ikR}\overset{N_{b}}{\underset{\mu }{\sum }}c_{\mu }^{\sigma }\left(k\right)\phi_{\mu }\left(r-r_{\mu }-R\right)$$ (4)

where *ϕ*_*μ*_(**r** − **r**_*μ*_ − **R**) denotes the *μ*-th NAO centering at **r**_*μ*_ within a lattice translation vector **R**, $c_{\mu }^{\sigma }\left(k\right)$ is the expansion coefficient, and *N* is the number of primitive unit cells under the Born-von Kármán (BvK) periodic boundary conditions. Within NAOs, the HFX matrix for the self-consistent field (SCF) calculations can be written as

$${\left[H_{x}^{\sigma ,HF}\right]}_{\mu \kappa }^{G}=-\underset{\nu \lambda }{\sum }\underset{N,H}{\sum }P_{\nu \lambda }^{\sigma ,H-N}\left(\mu^{0}\nu^{N}|\kappa^{G}\lambda^{H}\right)$$ (5)

and the corresponding HFX energy can be obtained by

$$E_{x}^{\text{HF}}=-\frac{1}{2}\underset{\sigma }{\sum }\underset{\mu \nu \kappa \lambda }{\sum }\underset{G,N,H}{\sum }P_{\mu \kappa }^{\sigma ,G}P_{\nu \lambda }^{\sigma ,H-N}\left(\mu^{0}\nu^{N}|\kappa^{G}\lambda^{H}\right)$$ (6)

where the subscripts of Greek letters {*μ*, *ν*, *κ*, *λ*} label NAOs, the superscript **0** represents the reference primitive unit cell, while **G**, **N**, and **H** represent extended unit cells in the BvK supercells. The $P_{\mu \kappa }^{\sigma ,G}$ is the spin density matrix element, which can be obtained by an integration of the expanded coefficients in the BZ

$$P_{\mu \kappa }^{\sigma ,G}=\underset{j}{\sum }\int_{\text{BZ}}c_{\mu ,j}^{\sigma *}\left(k\right)c_{\kappa ,j}^{\sigma }\left(k\right)\theta \left(\epsilon_{F}-\epsilon_{j}^{\sigma }\left(k\right)\right)e^{ikG}\;dk$$ (7)

where *θ* represent the step function, *ϵ*_*F*_ is the fermi energy and $\epsilon_{j}^{\sigma }\left(k\right)$ is the *j*-th eigenvalue at **k**. For hybrid functional calculations with NAOs, the main bottleneck is the evaluation of ERIs

$$\begin{array}{c} \left(\mu^{0}\nu^{N}|\kappa^{G}\lambda^{H}\right)=\iint \phi_{\mu }^{0}\left(r\right)\phi_{\nu }^{N}\left(r\right)\hat{v}\left(r,r^{\prime }\right)\phi_{\kappa }^{G}\left(r^{\prime }\right)\phi_{\lambda }^{H}\left(r^{\prime }\right)\;dr\;dr^{\prime } \end{array}$$ (8)

### 2.2 Analytical gradients of HFX energy

Since the NAOs are dependent of atomic positions, in hybrid functional calculations we must additionally calculate the HFX contribution to atomic forces. TheHFX forces acting on the *I*-th atom can be directly obtained from the negative gradients of the HFX energy with respect to atomic position **R**_*I*_

$$\begin{array}{cc} F_{I}^{\text{HF}} & =-\frac{\partial E_{x}^{\text{HF}}}{\partial R_{I}}\\ & =\;\underset{\sigma }{\sum }\underset{\mu \kappa }{\sum }\frac{\partial P_{\mu \kappa }^{\sigma ,G}}{\partial R_{I}}\underset{\nu \lambda }{\sum }\underset{N,H}{\sum }P_{\nu \lambda }^{\sigma ,H-N}\left(\mu^{0}\nu^{N}|\kappa^{G}\lambda^{H}\right)\\ & \;+\frac{1}{2}\underset{\sigma }{\sum }\underset{\mu \nu \kappa \lambda }{\sum }\underset{G,N,H}{\sum }P_{\mu \kappa }^{\sigma ,G}P_{\nu \lambda }^{\sigma ,H-N}\frac{\partial \left(\mu^{0}\nu^{N}|\kappa^{G}\lambda^{H}\right)}{\partial R_{I}} \end{array}$$ (9)

Note that the first term in Eq. 9 can be rewritten as

$$\underset{\mu \kappa }{\sum }\frac{\partial P_{\mu \kappa }^{\sigma ,G}}{\partial R_{I}}\underset{\nu \lambda }{\sum }\underset{N,H}{\sum }P_{\nu \lambda }^{\sigma ,H-N}\left(\mu^{0}\nu^{N}|\kappa^{G}\lambda^{H}\right)=-\underset{\mu \kappa }{\sum }{\left[H_{x}^{\sigma ,HF}\right]}_{\mu \kappa }^{G}\frac{\partial P_{\mu \kappa }^{\sigma ,G}}{\partial R_{I}}$$ (10)

which is automatically included in the orthogonalization force due to the non-orthonormality of the NAO basis set (Soler et al., 2002; Li et al., 2016). For periodic systems, the orthogonalization force is given by

$$F_{I}^{\text{orth}}=-\underset{\sigma }{\sum }\underset{\mu \kappa }{\sum }H_{\mu \kappa }^{\sigma ,G}\frac{\partial P_{\mu \kappa }^{\sigma ,G}}{\partial R_{I}}=\underset{\sigma }{\sum }\underset{\mu \kappa }{\sum }E_{\mu \kappa }^{\sigma ,G}\frac{\partial S_{\mu \kappa }^{G}}{\partial R_{I}}.$$ (11)

where $S_{\mu \lambda }^{G}=\left\langle \phi_{\mu }^{0}|\phi_{\lambda }^{G}\right\rangle$ is the overlap matrix element, and $E_{\mu \lambda }^{\sigma ,G}$ is the energy-density matrix element given by

$$E_{\mu \kappa }^{\sigma ,G}=\underset{j}{\sum }\int_{\text{BZ}}c_{\mu ,j}^{\sigma *}\left(k\right)c_{\kappa ,j}^{\sigma }\left(k\right)\epsilon_{j}^{\sigma }\left(k\right)e^{ikG}\;dk$$ (12)

where $\epsilon_{j}^{\sigma }\left(k\right)$ is the eigenstate energy.

Thus, we only need to deal with the second term

$$\begin{array}{cc} & \frac{1}{2}\underset{\sigma }{\sum }\underset{\mu \nu \kappa \lambda }{\sum }\underset{G,N,H}{\sum }P_{\mu \kappa }^{\sigma ,G}P_{\nu \lambda }^{\sigma ,H-N}\frac{\partial \left(\mu^{0}\nu^{N}|\kappa^{G}\lambda^{H}\right)}{\partial R_{I}}\\ & \;=\frac{1}{2}\underset{\sigma }{\sum }\underset{\mu \nu \kappa \lambda }{\sum }\underset{G,N,H}{\sum }P_{\mu \kappa }^{\sigma ,G}P_{\nu \lambda }^{\sigma ,H-N}\\ & \;\;\times \left(\frac{\partial \phi_{\mu }^{0}}{\partial R_{I}}\phi_{\nu }^{N}|\phi_{\kappa }^{G}\phi_{\lambda }^{H}\right)+\left(\phi_{\mu }^{0}\frac{\partial \phi_{\nu }^{N}}{\partial R_{I}}|\phi_{\kappa }^{G}\phi_{\lambda }^{H}\right)+\left(\phi_{\mu }^{0}\phi_{\nu }^{N}|\frac{\partial \phi_{\kappa }^{G}}{\partial R_{I}}\phi_{\lambda }^{H}\right)+\left(\phi_{\mu }^{0}\phi_{\nu }^{N}|\phi_{\kappa }^{G}\frac{\partial \phi_{\lambda }^{H}}{\partial R_{I}}\right) \end{array}$$ (13)

in which the ERI derivatives have to be evaluated properly. Since each ERI may have four different centers, a maximum of 12 differentials is required. Therefore, the calculation of HFX forces is formally more troublesome than that of HFX matrix, and a poor implementation will decrease the overall performance.

## 3 Methodology

### 3.1 NAO2GTO scheme

A normalized NAO for atom *I* located at **R**_*I*_ is defined as the product of a numerical radial function and a real regular solid harmonic (Soler et al., 2002)

$$\phi_{\mathrm{Ilm\zeta}}^{\text{NAO}}\left(r\right)=\phi_{Il\zeta }^{\text{NAO}}\left(r_{I}\right)\left[r_{I}^{l}Y_{lm}\left(\theta ,\varphi \right)\right]$$ (14)

where **r**_*I*_ = **r** − **R**_*I*_, *r*_*I*_ = |**r**_*I*_|, *l* and *m* label the angular and magnetic momentum quantum numbers, respectively. In multiple-*ζ* bases, *ζ* labels different basis with the same quantum numbers (*l*, *m*) but different radial shapes. For simplicity, we will omit the index *ζ* later. The numerical radial function involves a normalization factor *N* (*l*, *α*) and is numerically tabulated in a linear radial mesh. $\left[r_{I}^{l}Y_{lm}\left(\theta ,\varphi \right)\right]$ also includes its individual normalization factor. NAOs are strictly localized in real space, which provides greater convenience for linear-scaling DFT calculations. However, the evaluation of ERIs over NAOs in real space is computationally expensive, which will introduce a big prefactor in linear-scaling HFX calculations (Shang et al., 2010).

In the LCAO framework, GTOs are by far the most commonly used basis functions to represent the molecular orbitals. This preference is mainly due to the analytical properties of GTOs, which allow efficient evaluation of ERIs for (post-)Hartree-Fock calculations. A normalized primitive spherical harmonic GTO is defined as

$$\phi_{\mathrm{Ilm\alpha}}^{\text{GTO}}\left(r\right)=N\left(l,\alpha \right)\exp\left(-\alpha r_{I}^{2}\right)\left[r_{I}^{l}Y_{lm}\left(\theta ,\varphi \right)\right]$$ (15)

where *α* is the orbital exponent, and *N* (*l*, *α*) is the normalization factor over the radial coordinates

$$N\left(l,\alpha \right)={\left[\frac{2^{2l+3}\left(l+1\right)!\alpha^{l+3/2}}{\left(2l+2\right)!\pi^{1/2}}\right]}^{1/2}$$ (16)

It is worth noting that most efficient algorithms for the evaluation of ERIs are based on Cartesian primitive GTOs

$$G_{l\alpha }\left(r\right)=N\left(l_{x},l_{y},l_{z},\alpha \right){\left(x-R_{x}\right)}^{l_{x}}{\left(x-R_{y}\right)}^{l_{y}}{\left(z-R_{z}\right)}^{l_{z}}\exp\left[-\alpha {\left(r-R_{I}\right)}^{2}\right]$$ (17)

where *l* = *l*_*x*_ + *l*_*y*_ + *l*_*z*_ labels the angular-momentum quantum number, **R**_*I*_ = (*R*_*x*_, *R*_*y*_, *R*_*z*_) is the orbital center, and *N* (*l*_*x*_, *l*_*y*_, *l*_*z*_, *α*) is the normalization factor

$$N\left(l_{x},l_{y},l_{z},\alpha \right)=\left[{\left(\frac{2}{\pi }\right)}^{3/4}\frac{2^{l}\alpha^{\left(2l+3\right)/4}}{{\left[\left(2l_{x}-1\right)‼\left(2l_{y}-1\right)‼\left(2l_{z}-1\right)‼\right]}^{1/2}}\right]$$ (18)

For a shell of angular momentum *l*, there will be 2*l* + 1 spherical GTOs, but (*l* + 1) (*l* + 2)/2 Cartesian GTOs. The transformation between normalized spherical and Cartesian GTOs is required with a transformation matrix *c* (*l*, *m*, *l*_*x*_, *l*_*y*_, *l*_*z*_) given by Schlegel and Frisch (Schlegel and Frisch, 1995).

In order to obtain ERIs efficiently, we can represent the NAOs in terms of a linear combination of spherical primitive GTOs, and then calculate the ERIs analytically by calling available libraries for GTO-based integrals (e.g. LIBINT (Valeev and Fermann, 2014)). This scheme is called NAO2GTO, which in principle is quite similar to the minimal STO-*n*G basis set used in the quantum chemistry community. In the NAO2GTO scheme, the numerical radial function of each NAO is fitted as a linear combination of different Gaussians,

$$\phi_{l}^{\text{NAO}}\left(r\right)≃\phi_{l}^{\text{CGTO}}\left(r\right)=\overset{M}{\underset{i=1}{\sum }}D_{i}\exp\left(-\alpha_{i}r^{2}\right)$$ (19)

where *M* is the number of Gaussians, *α*_*i*_ and *D*_*i*_ are the contraction coefficient and exponent, respectively, similar to contracted GTOs (CGTOs).

### 3.2 Replace NAOs with discretized CGTOs

If the NAO2GTO fitting is strictly accurate, the fitted orbitals will be automatically normalized because of the normalization of NAOs. Once there is a non-negligible fitting error, the fitted orbitals will not be normalized. Since normalization factors between NAOs and CGTOs are different, the following approximation will no longer hold

$$\phi_{lm}^{\text{NAO}}\left(r\right)\neq \phi_{lm}^{\text{CGTO}}=N\left(l,\alpha ,D\right)\overset{M}{\underset{i=1}{\sum }}D_{i}\exp\left(-\alpha_{i}r^{2}\right)r^{l}Y_{lm}\left(\theta ,\varphi \right)$$ (20)

with a normalization factor

$$N\left(l,\alpha ,D\right)={\left[\frac{\left(2l+2\right)!\pi^{1/2}}{2^{2l+3}\left(l+1\right)!}\overset{M}{\underset{i,j=1}{\sum }}\frac{D_{i}D_{j}}{{\left(\alpha_{i}+\alpha_{j}\right)}^{l+3/2}}\right]}^{-1/2}$$ (21)

where *α* = {*α*_*i*_} and **D** = {*D*_*i*_}. In practice, it is difficult to achieve an exact NAO2GTO fitting with a small number of GTOs, such as *M* = 3–6. Then, the NAO2GTO fitting will inevitably introduce the ERI errors between original NAOs and fitted CGTOs. In some cases, such errors can even invalidate the final results. In our previous work, we employed the fitted CGTOs to approximately evaluate the ERIs for the HFX term while retaining original NAOs for pure DFT parts. For most systems, we have found that a self-consistent convergence problem often arises in hybrid functional calculations even with high-precision fitting.

To eliminate the fitting errors properly, here we replace original NAOs with the fitted and discretized CGTOs, which is done as the following steps:

• Perform a less rigorous NAO2GTO fitting for each NAO to obtain a set of CGTOs according to Eq. 19;
• Calculate the normalization constant *N* (*l*, *α*, **D**) for each CGTO;
• Calculate the cutoff radius for each CGTO;
• Numerically tabulate the radial function of each CGTO multiplied by *N* (*l*, *α*, **D**).

Note that the fitted CGTOs will give larger cutoff radii compared to the original NAOs. Therefore, we need to feed the values inside a new cutoff radius back to the radial function, beyond which all values are equal to 0.

### 3.3 Evaluate ERIs and their derivatives with CGTOs

With the auxiliary CGTOs, one shell set of contracted ERIs (*ab*|*cd*) can be calculated by

$$\left(ab|cd\right)=\overset{K}{\underset{k}{\sum }}\overset{L}{\underset{l}{\sum }}\overset{M}{\underset{m}{\sum }}\overset{N}{\underset{n}{\sum }}C_{ak}C_{bl}C_{cm}C_{dn}\left[a_{k}b_{l}|c_{m}d_{n}\right]$$ (22)

with

$$C_{ak}=D_{ak}c\left(a,m,a_{x},a_{y},a_{z}\right)$$ (23)

where *a* denotes the shell orbital with angular momentum *a* = *a*_*x*_ + *a*_*y*_ + *a*_*z*_, *k* is the index of *K* GTOs, and [*a*_*k*_ *b*_*l*_|*c*_*m*_ *d*_*n*_] represents a set of primitive ERIs over primitive Cartesian GTOs, in which (2*a* + 1) (2*b* + 1) (2*c* + 1) (2*d* + 1) primitive and contacted ERIs are calculated at once.

Since a primitive ERI contain four centers of **A**, **B**, **C**, and **D**, its first-order derivatives should have the following 12 terms

$$\frac{\partial \left[ab|cd\right]}{\partial A_{i}},\;\frac{\partial \left[ab|cd\right]}{\partial B_{i}},\;\frac{\partial \left[ab|cd\right]}{\partial C_{i}},\;\frac{\partial \left[ab|cd\right]}{\partial D_{i}}\;\;\;i\in \left\{x,y,z\right\}.$$ (24)

but only 9 derivatives are required because of the translational invariance

$$\frac{\partial \left[ab|cd\right]}{\partial A_{i}}+\frac{\partial \left[ab|cd\right]}{\partial B_{i}}+\frac{\partial \left[ab|cd\right]}{\partial C_{i}}+\frac{\partial \left[ab|cd\right]}{\partial D_{i}}=0$$ (25)

Analogously, the first-order derivatives of contracted ERIs can also be evaluated from the primitive ones, which are actually a linear combination of higher and lower angular momentum ERIs

$$\frac{\partial }{\partial A_{i}}\left[ab|cd\right]=2\alpha \left[\left(a+1_{i}\right)b|cd\right]-a_{i}\left[\left(a-1_{i}\right)b|cd\right]$$ (26)

We obtain the primitive ERIs and their derivatives from the LIBINT library, (Valeev and Fermann, 2014) which implements efficient recursive schemes based on the Obara-Saika method (Obara and Saika, 1986) together with the Head-Gordon-Pople (Head-Gordon and Pople, 1988) and Hamilton-Lindh (Hamilton and Schaefer, 1991; Lindh et al., 1991) variations. We also consider the eight-fold permutational symmetry of ERIs with NAOs, which for periodic systems is given by

$$\begin{array}{cc} & \left(\mu^{0}\nu^{N}|\kappa^{G}\lambda^{H}\right)=\left(\mu^{0}\nu^{N}|\lambda^{H}\kappa^{G}\right)=\left(\nu^{0}\mu^{-N}|\kappa^{G-N}\lambda^{H-N}\right)=\left(\nu^{0}\mu^{-N}|\lambda^{H-N}\kappa^{G-N}\right)\\ & =\left(\kappa^{0}\lambda^{N-G}|\mu^{-G}\nu^{N-G}\right)=\left(\kappa^{0}\lambda^{N-G}|\nu^{N-G}\mu^{-G}\right)=\left(\lambda^{0}\kappa^{G-H}|\mu^{-H}\nu^{N-H}\right)=\left(\lambda^{0}\kappa^{G-H}|\nu^{N-H}\mu^{-H}\right) \end{array}$$ (27)

In this way, we only need to handle about 1/8 of ERIs and their derivatives. Furthermore, the translational invariance also gives $\frac{\partial \left(\mu \mu |\mu \mu \right)}{\partial R_{\mu }}=0$ , which means that we can ignore the ERI derivatives if four orbitals have the same center.

### 3.4 Integral screening

In practice calculations, most of the ERIs and their derivatives have no significant contributions to the HFX matrix and forces, which can be omitted by using integral screening techniques. Thus, integral screening is essential for reducing the computational cost, which should be able to provide an easy-to-estimate upper bound for ERIs. We have previously employed several ERI screening techniques to obtain an efficient and linear-scaling HFX calculation (Shang et al., 2011; Qin et al., 2015). For the calculation of HFX forces, however, integral screening based on the upper bound of ERI derivatives is not a good choice. The reason is that, according to the Schwarz inequality (Häser and Ahlrichs, 1989), the upper bound of $\left(\frac{\partial \mu }{\partial R_{I}}\nu |\lambda \sigma \right)$ requires a relatively expensive calculation of $\left(\frac{\partial \mu }{\partial R_{I}}\nu |\frac{\partial \mu }{\partial R_{I}}\nu \right)$ (Horn et al., 1991), which does not appear in the ERI derivatives. Alternatively, we can use the same screening techniques based on the upper bound of ERIs as done in the construction of the HFX matrix. That is, if an ERI is skipped during the calculation of the HFX energy, its derivatives should also be neglected for the calculation of HFX forces. Here, we describe all the screening techniques we have employed to compute the HFX forces. For simplicity, we omit the superscripts of **0**, **G**, **N**, and **H** later, and the indices {*μ*, *ν*, *κ*, *λ*} label shell orbitals in the following.

#### 3.4.1 Schwarz screening with prametrized screening functions

The first integral screening is based on Cauthy-Schwarz inequality

$$|\left(\mu \nu |\kappa \lambda \right)|\leq |\left(\mu \nu |\mu \nu \right){|}^{1/2}|\left(\kappa \lambda |\kappa \lambda \right){|}^{1/2}$$ (28)

which gives a rigorous upper bound for an ERI or a set of ERIs. The Schwarz screening actually takes advantage of the exponential decay of the orbital-pair charge distributions Ω_*μν*_(**r** − **P**) = *χ*_*μ*_(**r** − **R**_*μ*_)*χ*_*ν*_(**r** − **R**_*ν*_) to decrease the total number of ERIs to be considered from $O\left(N^{4}\right)$ to $O\left(N^{2}\right)$ . To establish a straightforward Schwarz-screening procedure, we need to calculate and store two-center integrals as the screening matrix in the four-index (*μ*, *ν*, *κ* and *λ*) loop. However, this treatment is not efficient for large systems in which a large screening matrix is needed. Furthermore, it is inconvenient to employ the Schwarz screening for primitive ERIs since each shell quartet also requires calculating and storing its own screening matrix. In fact, it has been observed by Guidon et al. (2009) that the logarithm of a two-center ERI can be approximated as a quadratic function at a relatively large two-center distance *R*_*μν*_ between orbitals *μ* and *ν*

$$\log|\left(\mu \nu |\mu \nu \right)|\left(R_{\mu \nu }\right)|\approx t_{2}R_{\mu \nu }^{2}+t_{0}.$$ (29)

**FIGURE 1:**
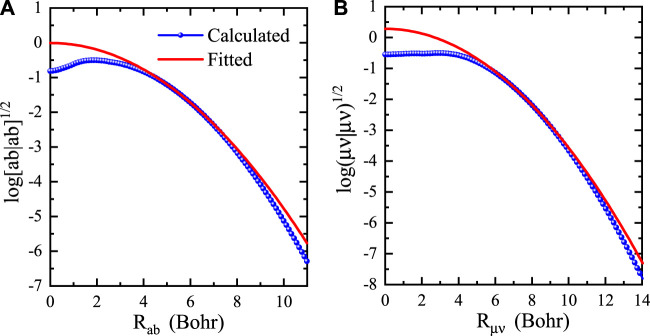
Logarithm of two-center integrals (blue solids) and fitting functions (red lines) as a function of the two-center distance for **(A)** primitive and **(B)** contracted integrals of *p*-type and *d*-type Si orbitals.

These quadratic functions only depend on the two-center distance *R*_*μν*_ but have different parameters *t*_0_ and *t*_2_ for different types of orbitals. Thus, we can use a set of quadratic functions (screening functions) instead of the screening matrix to estimate the upper bounds of both primitive and contracted ERIs. We obtain the fitting parameters *t*_0_ and *t*_2_ by minimizing an asymmetric penalty function (Guidon et al., 2009),

$$\underset{i}{\sum }k\left(\Delta_{i}\right)\Delta_{i}^{2}.$$ (30)

at a radial grid of $R_{\mu \nu }^{i}$ with the maximum distance $R_{\mu \nu }=R_{c}^{\mu }+R_{c}^{\nu }$ , and the error is defined as

$$\Delta_{i}=\log|\left(\mu \nu |\mu \nu \right)\left(R_{\mu \nu }\right)|-\left(t_{2}R_{\mu \nu }^{2}+t_{0}\right)$$ (31)

and

$$k\left(\Delta_{i}\right)=\left\{\begin{array}{cc} 1\; & if\;\;\Delta_{i}<0;\\ 10^{4}\; & if\;\;\Delta_{i}\geq 0. \end{array}\right.$$ (32)

where the choice of *k* (Δ_*i*_) ensures the fitted value at $R_{\mu \nu }^{i}$ is always not less than the true one. In Figure 1, we plot the fitting results for both primitive and contracted two-center integrals. It can be seen that the fitted value for each two-center distance is never less than the true one, indicating that the screening function can be approximately used as an upper bound in the Schwarz screening. As a result, we only need to fit the screening functions and store the fitting parameters for each type of shell pairs in advance, as shown in Algorithm 1 (Step 1).

Algorithm 1 Flowchat of density matrix weighted Schwarz screening for HFX forces.

1: Step 1: Fit screening functions ⊳ For different types of shell orbitals
2: **for** is and js ∈ *N*_species_ **do** ⊳ Atomic species *N*_species_
3: **for** *μ* ∈ is, *ν* ∈ js **do**
4: **for** *a* ∈ *μ*, *b* ∈ *ν* **do**
5: *R*_*ab*_ = *R*_*a*_ + *R*_*b*_
6: Interpolate *R*_*i*_ and calculate[*ab*|*ab*](*R*_*i*_)
7: Fit $\log|{\left[ab|ab\right]}^{1/2}\left(R_{ab}\right)|\approx t_{2}^{ab}R_{ab}^{2}+t_{0}^{ab}$ .
8: **end for**
9: *R*_*μν*_ = max{*R*_*μν*_, *R*_*ab*_}
10: Interpolate *R*_*i*_ and calculate (*μν*|*μν*)(*R*_*i*_)
11: Fit $\log|{\left(\mu \nu |\mu \nu \right)}^{1/2}\left(R_{\mu \nu }\right)|\approx t_{2}^{\mu \nu }R_{\mu \nu }^{2}+t_{0}^{\mu \nu }$

12: **end for**
13: **end for**
14: Step 2: Build shell-pair lists ⊳ For all shell orbitals
15: **for** *μ* = 1, *N*_b_ **do**
16: **for** *ν* = 1, *μ* **do**
17: $t_{2}^{\text{max}}=\max\left\{t_{2}^{\text{max}},t_{2}^{\mu \nu }\right\}$

18: $t_{0}^{\text{max}}=\max\left\{t_{0}^{\text{max}},t_{0}^{\mu \nu }\right\}$

19:  **if** $R_{\mu \nu }\leq R_{c}^{\mu }+R_{c}^{\nu }$ and $\left(t_{2}^{\mu \nu }R_{\mu \nu }+t_{0}^{\mu \nu }\right)+\left(t_{2}^{\text{max}}R_{\mu \nu }+t_{0}^{\text{max}}\right)>\log\epsilon_{\text{Schwarz}}$ **then**
20:  Add *μ*, *ν* to shell-pair list_*μν*_
21:  **end if**
22:  **end for**
23:  **end for**
24:  Step3: Compute HFX forces
25:  **for** (*μ*, *ν*) ∈ list_*μν*_ **do**
26:  **for** (*κ*, *λ*) ∈ list_*κλ*_ **do**
27: $P_{\text{max}}=2\times \max\left\{\left|P_{\mu \kappa }^{\sigma }|\times |P_{\nu \lambda }^{\sigma }|,|P_{\mu \lambda }^{\sigma }|\times |P_{\nu \kappa }^{\sigma }\right|\right\}$

28:  **if** $\log P_{\text{max}}+t_{2}^{\mu \nu }R_{\mu \nu }+t_{0}^{\mu \nu }+t_{2}^{\kappa \lambda }R_{\kappa \lambda }+t_{0}^{\kappa \lambda }>\log\epsilon_{\text{Schwarz}}$ **then**
29:   **for** (*k*, *l*, *m*, *n*) ∈ (*K*, *L*, *M*, *N*) **do**
30: $C_{\text{max}}=\max\left\{C_{\text{max}},C_{a_{k}}C_{b_{l}}C_{c_{m}}C_{d_{n}}\right\}$

31:   **if** $\log P_{\text{max}}+\log C_{\text{max}}+\left(t_{2}^{ab}R_{ab}+t_{0}^{ab}\right)+\left(t_{2}^{cd}R_{cd}+t_{0}^{cd}\right)>\log\epsilon_{\text{Schwarz}}$ **then**
32:    **if** Far-field SR ERIs and *P*_max_ × *C*_max_ × [*ab*|*cd*]_SR_ > *ϵ*_Far-filed_ **then**
33:     Call LIBINT to calculate primitive ERI derivatives
34:    **end if**
35:   **end if**
36:   Calculate contracted ERI derivatives
37:  **end for**
38:   Calculate HFX forces according to Eq. 13
39:  **end if**
40: **end for**
41: **end for**

#### 3.4.2 Far-field distance screening

The distance screening proposed by Izmaylov et al. (Izmaylov et al., 2006) further takes into account the decay of SR ERIs with respect to the distance *R*_PQ_ between two charge distribution centers **P** and **Q**. According to the multipole expansion, the primitive ERIs can be divided into near-field and far-field parts by (Burant et al., 1996)

$$R_{\text{PQ}}\geq {\tilde{R}}_{P}+{\tilde{R}}_{Q},\;\;\tilde{R}=int\left[{\left(2\alpha \right)}^{-1/2}{erfc}^{-1}\left(\epsilon \right)\right]+1,$$ (33)

where *ϵ* is a threshold that defines the spatial range of a distribution $\tilde{R}$ . The far-field SR ERIs have the following approximation

$${\left[ab|cd\right]}_{\text{SR}}\approx K_{ab}K_{cd}\frac{erfc\left(\theta_{\omega }^{1/2}R_{\text{PQ}}\right)}{R_{\text{PQ}}}$$ (34)

with

$$K_{ab}=\frac{\sqrt{2}\pi^{5/4}}{\alpha +\beta }\exp\left[-\frac{\alpha \beta }{\alpha +\beta }{\left(A-B\right)}^{2}\right]$$ (35)

Thus, in HSE calculations we can employ the distance screening based on Eq. 34 to screen out far-field primitive ERIs.

#### 3.4.3 NAO screening

The NAOs are strictly localized in real space, so the ERIs over NAOs will be strictly zero and negligible if two shell orbitals *μ* and *ν* (or *κ* and *λ*) do not overlap with $R_{\mu \nu }>R_{c}^{\mu }+R_{c}^{\nu }$ . To reduce the number of four-index loops, as shown in Algorithm 1 (Step 2), we first construct two shell-pair lists (list_*μν*_ and list_*κλ*_) by taking into account the locality of NAOs, which is done prior to entering the calculation of HFX forces.

On the other hand, both the Hamiltonian and density matrices in pure DFT calculations exhibit a sparse pattern determined by the locality of NAOs. The matrix element *H*_*μκ*_ is non-zero only when the orbitals *μ* and *κ* directly overlap each other or indirectly overlap through a non-local pseudopotential projector. For hybrid functional calculations, the HFX matrix can also be stored in the same sparse format as that of pure DFT. According to Eq. 5, the NAO screening can screen out the ERIs for all shell pairs (*μ*, *κ*), (*μ*, *λ*), (*ν*, *κ*), and (*ν*, *λ*) do not overlap when considering the full ERI symmetry.

#### 3.4.4 Density matrix screening

Our initial implementation of hybrid functionals is based on a non-direct SCF scheme, in which the ERIs are precalculated and stored in memory or disk with the above integral screening approaches (Shang et al., 2011). However, this scheme has a storage bottleneck and is not efficient for large systems since it does not exploit the sparse density matrix for integral screening. In fact, the ERIs are coupling with the density matrix in Eq. 5 and Eq. 6, which means that a large ERI (*μν*|*κλ*) may also be negligible if the density matrix elements *P*_*μκ*_ and *P*_*νλ*_ are fairly small. The integral screening techniques to achieve linear scaling HFX calculations are linked closely to the sparse density matrix, such as the ONX (Burant et al., 1996; Schwegler and Challacombe, 1996) and LinK (Schwegler et al., 1997) algorithms. It should be pointed out that the NAO screening partially takes into account the sparsity of the density matrix, so it can also lead to a linear scaling HFX calculation (Shang et al., 2011).

In order to improve the screening efficiency, we employ the density-matrix- based screening approach combined with a direct SCF scheme to calculate ERIs on-the-fly at each SCF iteration, which avoids the usage of memory for ERIs. The density matrix screening is to introduce the density matrix in the Schwarz and distance screening procedures. Considering the full ERI symmetry, the density matrix weighted Schwarz screening for building the HFX matrix is given by

$$\max\left\{\left|P_{\mu \kappa }^{\sigma }|,|P_{\mu \lambda }^{\sigma }|,|P_{\nu \kappa }^{\sigma }|,|P_{\nu \lambda }^{\sigma }\right|\right\}\times |\left(\mu \nu |\mu \nu \right){|}^{1/2}|\left(\kappa \lambda |\kappa \lambda \right){|}^{1/2}\leq \epsilon_{\text{Schwarz}}$$ (36)

where the initial density matrix can be obtained from a post-PBE calculation for the first SCF step. The HFX forces are calculated by direct differentiation of the HFX energy after the SCF convergence, thus the ERI derivatives can be screened out if the corresponding ERIs have a negligible contribution to the HFX energy. Following Guidon et al. (Guidon et al., 2008), we adopt the following screening criterion for the calculation of HFX forces

$$2\times \max\left\{\left|P_{\mu \kappa }^{\sigma }|\times |P_{\nu \lambda }^{\sigma }|,|P_{\mu \lambda }^{\sigma }|\times |P_{\nu \kappa }^{\sigma }\right|\right\}\times |\left(\mu \nu |\mu \nu \right){|}^{1/2}|\left(\kappa \lambda |\kappa \lambda \right){|}^{1/2}\leq \epsilon_{\text{Schwarz}}.$$ (37)

where the factor 2 is derived from the double contributions of ERIs to the first term of HFX forces in Eq. 9. Our density matrix weighted Schwarz screening for the calculation of HFX forces is shown in Algorithm 1 (Step 3). Since the products of converged density matrix elements are used, the calculation of the HFX forces will be more faster than the construction of the HFX matrix at each SCF step.

### 3.5 Parallelization strategy

With the rapid development of computer clusters and supercomputers, high-performance computing (HPC) is now essential for program design. In the parallel implementation of HFX forces, the key is to distribute the calculation of ERI derivatives across different central processing unit (CPU) cores. Of course, a straightforward way is to evenly distribute all shell quartets on each processor. However, the total amount of shell quartets is unknown until the integral screening is finished. Furthermore, the computational cost for different types of shell quartets may be very different. For instance, (*dd*|*dd*) with higher order angular momentum may be hundreds of times more expensive than (*ss*|*ss*), which also may cause serious load imbalance. On the other hand, the distribution of the density matrix may also introduce additional communication when considering the full ERI symmetry. Therefore, load balancing and minimizing communication overhead are essential considerations in the parallel design of HFX force calculation.

For massively parallel computing, a better choice is to employ the master-worker dynamic parallel scheme, which can yield very high load balance and parallel efficiency. We have established a master-worker parallel scheme for the calculation of HFX forces as that for the construction of HFX matrix based on the dynamic parallel distribution algorithm (Shang et al., 2020). In this scheme, one message passing interface (MPI) process is designated as the master to manage the distribution of the shell quartets, while the remaining processes act as the workers responsible for integral evaluation. The computational task corresponding to the total amount of shell quartets is obtained by multiplying the size of shell-pair lists. Then, the shell quartets are assigned by the master to the worker processes in batches by request at a time. Each worker process requests individual and batched shell quartets from the master, and computes the ERI derivatives and HFX forces with integral screening. Once the current tasks are completed, the worker process continues to request new shell quartets until there are no tasks left. In order to further reduce the data communication for the density matrix, we replicate the global density matrix on each individual MPI process. Unlike the HFX matrix construction, the calculation of HFX forces does not require a MPI_ALLREDUCE operation, thus global communication can be avoided.

In recent years, heterogeneous architectures with dedicated accelerators have become increasingly available in modern HPC systems. As one of the most widely used accelerators, graphics processing unit (GPU) is designed specifically for handling massive parallelism and performing multiple computational tasks simultaneously. Nowadays, numerous quantum chemistry software packages have been equipped with GPU support, in particular, to accelerate the evaluation of ERIs and their subsequent contraction for constructing the HFX matrix (Ufimtsev and Martinez, 2008, 2009; Barca et al., 2020). The GPU acceleration can be accomplished by mapping ERIs onto GPU threads, using either a one-block-one-contracted-integral algorithm or a one-thread-one-contracted-integral algorithm (Ufimtsev and Martinez, 2008). Regarding the master-worker parallel distribution, we can set an appropriate batch size and map the batched ERIs of a request across the GPU blocks or threads, which depends on the available resources on the GPU associated with each worker process. Therefore, our parallelization strategy presented above could also be extended to CPU-GPU heterogeneous parallelism, even though such an extension is not covered in the present work.

## 4 Results and discussion

In this section, we focus on demonstrating the numerical accuracy and efficiency of our implementation for HFX force calculations of periodic systems in the HONPAS package (Qin et al., 2015; Qin et al., 2020a). The norm-conserving PBE pseudopotentials of the Troullier-Martins type (Troullier and Martins, 1991) are used to represent the core-valence interaction. The pseudopotential for Ti includes semicore states (3*s* and 3*p*) in the valence. The NAOs are generated using default parameters or are optimized using the simplex utility in SIESTA, and the double-*ζ* plus polarization (DZP) basis set is employed. 5, 4, and 3 GTOs, namely 543, are used for *s*-, *p*-, and *d*-type NAOs of B, C, O, Si, and P, while a 544 fitting is used for Ti. The real-space mesh cutoff for all systems is set to 250 Ry. The tolerance of density matrix for the SCF convergence and the force tolerance in coordinate optimization are set to the values of 10^–4^ and 0.01 eV/Å, respectively. After k-point convergence tests, the 8 × 8 × 8 Monkhorst-Pack k-point sampling in the BZ is chosen for bulk systems (Diamond, Si, SiC, BN, and BP).

### 4.1 Numerical accuracy and efficiency

#### 4.1.1 Fitted orbitals for NAOs

In this work, we first use a linear combination of several Gaussians to fit the tabulated radial function of NAOs based on the NAO2GTO scheme and take the renormalized CGTOs as the new numerical basis functions with a cutoff radius *R*_c_. Since the CGTOs decay exponentially in real space, we need to truncate them with a cutoff threshold defined as *ϕ* ^CGTO^ (*R*_c_) < *ϵ*_cut_. As illustrated in Table 1, a smaller threshold will result in a larger cutoff radius for each CGTO. When *ϵ*_cut_ is set to 10^–3^, the radius of a CGTO is slightly larger than that of its original NAO. However, a smaller value of *ϵ*_cut_ = 10^–7^ will yield 1.5–2 times larger cutoff radii. In practice, the cutoff radius also depends on the minimum fitting exponent *α*_min_, which is selected to be 0.15 in order to prevent the generation of too diffuse GTOs.

**TABLE 1:** The cutoff radii (in Bohr) of orbitals at a given threshold (*ϵ*_cut_) for silicon atom with the DZP basis set. *s*_1_ and *s*_2_ label the 1st-*ζ* and 2nd-*ζ s*-type orbitals, *p*_1_ and *p*_2_ label the 1st-*ζ* and 2nd-*ζ p*-type orbitals, and *d* denotes the polarized *d*-type orbital.

Orb.	*ϵ*_cut_	*s*_1_	*s*_2_	*p*_1_	*p*_2_	*d*
NAO		5.007	4.419	6.271	5.007	6.271
CGTO	10^–^^3^	5.172	4.557	8.481	6.093	7.789
	10^–^^4^	5.972	5.262	9.549	6.889	8.630
	10^–^^5^	6.677	5.883	10.500	7.595	9.385
	10^–^^6^	7.313	6.445	11.366	8.236	10.077
	10^–^^7^	7.900	6.961	12.167	8.829	10.720

We determine the cutoff radii by examining the ERI errors resulting from the truncation of CGTOs. We compare the ERIs for fitted CGTOs with different cutoff thresholds by using numerical and analytical integrations, respectively. As listed in Table 2, the maximum absolute error (Δ*E*_max_) of ERIs can be as less as 2.4 × 10^–6^ eV (1.76 × 10^–7^ Ry) if *ϵ*_cut_ = 10^–5^ is given. Therefore, we decide to choose *ϵ*_cut_ = 10^–5^ as the default cutoff threshold for all hybrid functional calculations. From Table 2, we can also observe that the calculated ERIs over original NAO and fitted CGTO differ by a maximum of 0.533 eV. Such a significant difference may render the hybrid functional calculation invalid if we use the fitted CGTOs for the HFX term while still relying on the original NAOs for other terms. To ensure the accuracy and reliability of the NAO2GTO scheme, we decide to apply the fitted CGTOs consistently across all components of the hybrid functional calculations. Specifically, we employ the analytical CGTOs for the HFX calculation while the numerically discretized CGTOs for other pure DFT calculations.

**TABLE 2:** Analytical (Analy.) and Numerical (Numer.) ERIs (in eV) at a given cutoff threshold (*ϵ*_cut_). The tested system is the silicon atom with the DZP basis set. *s*_1_ and *s*_2_ label the 1st-*ζ* and 2nd-*ζ s*-type orbitals, *p*_1_ and *p*_2_ label the 1st-*ζ* and 2nd-*ζ p*-type orbitals with *m* = −1, *d* label the polarized *d*-type orbitals with *m* = −2, Δ*E*_max_ is the maximum absolute error between numerical and analytical ERIs.

Method	*ϵ*_cut_	(*s*_1_ *s*_1_|*s*_1_ *s*_1_)	(*s*_2_ *s*_2_|*s*_2_ *s*_2_)	(*p*_1_ *p*_1_|*p*_1_ *p*_1_)	(*p*_2_ *p*_2_|*p*_2_ *p*_2_)	(*dd*|*dd*)	Δ*E*_max_
Analy.	CGTO	11.80152342	13.23662039	10.35761045	12.56349594	9.28132159	
Numer.	10^–3^	11.80001727	13.23633404	10.35760717	12.56348421	9.28132044	2.32 × 10^–3^
	10^–4^	11.80150036	13.23661751	10.35761042	12.56349583	9.28132172	6.41 × 10^–5^
	10^–5^	11.80152312	13.23662036	10.35761045	12.56349594	9.28132173	2.4 × 10^–6^
	10^–6^	11.80152342	13.23662039	10.35761046	12.56349595	9.28132173	1.4 × 10^–7^
	10^–7^	11.80152342	13.23662038	10.35761046	12.56349595	9.28132173	1.4 × 10^–7^
	Original NAO	11.80299940	13.23767035	10.37228773	12.57084510	8.74843515	5.33 × 10^–1^

#### 4.1.2 Integral screening

We then benchmark the numerical accuracy and efficiency of different screening methods for the Si crystal with HSE06 calculations. The lattice constant is chosen to be 5.43 Å, and the primitive unit cell containing two Si atoms is used. The Cartesian coordinates of two atoms are set to non-equilibrium positions of (0, 0, 0) and (1.3175, 1.3575, 1.3575), respectively, so that a relatively large value of atomic force in the *x* direction can be obtained. Table 3 shows the absolute errors (total energy, band gap, and atomic force) and the wall time for the calculation of HFX matrix and forces under different screening methods and thresholds. The reference values in the table are obtained using Schwarz-screening only with a threshold of *ϵ*_Schwarz_ = 10^–10^ (in Ry), in which the HFX time is taken from the last SCF step.

**TABLE 3:** Absolute errors (total energy Δ*E*_tot_ and band gap Δ*E*_g_ in eV, while atomic force Δ*F*_*x*_ in eV/Å) and wall time (in seconds) of different integral screening techniques for the Si crystal. All calculations are performed on 24 CPU cores.

	*ϵ*_Schwarz_	*ϵ*_Farfield_	Δ*E*_tot_	Δ*E*_g_	Δ*F*_*x*_	*T*_HFX_	*T*_Force_
Ref.	10^–^^10^	None	-213.752168	-1.1946	0.660689	1392.2	18829.6
A	10^–^^7^	None	4 × 10^–6^	0	1 × 10^–6^	509.5	6622.9
	10^–^^6^	None	7.0 × 10^–5^	0	1.0 × 10^–5^	333.7	4345.0
	10^–^^5^	None	7.71 × 10^–4^	1.2 × 10^–4^	1.79 × 10^–4^	209.9	2802.3
AB	10^–^^7^	10^–7^	4 × 10^–6^	0	0	410.0	5257.9
	10^–^^6^	10^–6^	7.2 × 10^–5^	0	1.3 × 10^–5^	271.2	3524.3
	10^–^^5^	10^–5^	7.77 × 10^–4^	1.2 × 10^–3^	1.86 × 10^–4^	171.0	2317.7
ABC	10^–^^7^	10^–7^	4 × 10^–6^	0	0	200.3	2595.3
	10^–^^6^	10^–6^	7.2 × 10^–5^	0	1.3 × 10^–5^	131.5	1666.5
	10^–^^5^	10^–5^	7.77 × 10^–4^	1.2 × 10^–3^	1.86 × 10^–4^	80.3	1043.0
ABCD	10^–^^7^	10^–7^	5.3 × 10^–5^	0	1.7 × 10^–5^	69.5	62.4
	10^–^^6^	10^–6^	7.08 × 10^–4^	4.0 × 10^–4^	6.96 × 10^–4^	37.0	18.8
	10^–^^5^	10^–5^	2.74 × 10^–3^	1.31 × 10^–2^	5.38 × 10^–3^	15.1	4.8

As can be seen from Table 3, both the numerical accuracy and computational cost can efficiently be controlled by the thresholds. The screening methods of Schwarz (A), far-field (B), and NAO (C) show almost the same errors in energy and force at given thresholds, while the density matrix screening (D) yields relatively larger errors. All absolute errors lie within the range of 10^−5^–10^−4^ (eV or eV/Å) when the thresholds are set to be 10^–6^ (Ry), which is then chosen as the global default threshold. For building the HFX matrix, we find that applying more screening methods of A, B, and C only leads to 1-2 speed-up, which can be improved by further including D. The calculation of HFX forces requires to evaluate the first-order derivatives of ERIs, which contain 12 components. Therefore, we can also see that the computational time of HFX forces under the same screening methods without the density matrix is about 12 times higher than that of HFX matrix. However, if the density matrix screening is involved, the computational cost of HFX forces can be reduced by nearly 2-3 orders of magnitude. In particular, the HFX force calculation can eventually be faster than the HFX matrix construction with the thresholds larger than 10^–7^. This dramatic improvement in efficiency can be attributed to two aspects: (1) the fully converged density matrix after the SCF iteration is more sparse; (2) the product of the sparse density matrix yields a smaller upper bound to filter out much more shell quartets.

**FIGURE 2:**
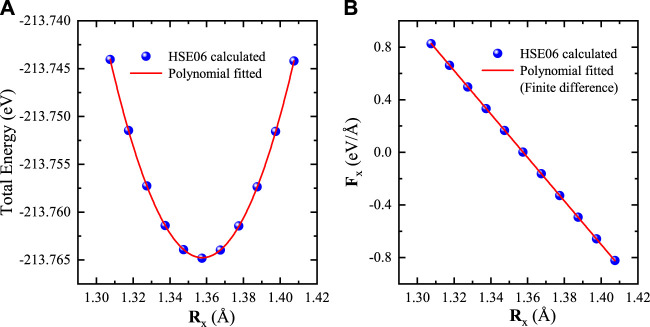
**(A)** Total energy and **(B)** atomic force as a function of Si coordinates. One atom is fixed at the origin, whereas the other atom is moved along the *x* direction. The curve shows a polynomial fitting of total energy, and its partial differential (the slope) is the numerical force **F**_*x*_ = *∂E*_*tot*_/*∂* **R**_*x*_ with the finite difference method.

We also compare the analytical and numerical gradients of total energy for the Si crystal. The numerical gradients are obtained by using the finite difference method, in which the first atom Si_1_ is fixed at the origin and the other atom Si_2_ located at (*x*, 1.3575, 1.3575) is moved along *x* direction. We perform a series of HSE06 calculations by varying the *x* coordinate of Si_2_ from 1.3075 to 1.4075 Å. As shown in Figure 2, the *x* component of analytical forces acting on Si_2_ are in very good agreement with the numerical differentiations of total energies. The maximum discrepancy between the analytical and numerical forces is less than 1.5 × 10^–3^ eV/Å, which also indicates that our implementation is correct.

#### 4.1.3 Lattice constant, bulk modulus, and band gap

Furthermore, we verify the reliability of our improved NAO2GTO scheme that replaces the NAOs with numerically discretized CGTOs. We calculate the equilibrium lattice constants *a*_0_, bulk moduli *B*_0_, and band gaps *E*_g_ for several typical semiconductors with HSE06, and compare them with experimental (Heyd and Scuseria, 2004) and other theoretical (Paier et al., 2006a; Levchenko et al., 2015) results. The equilibrium lattice constants and bulk moduli are determined by fitting energy-volume (E-V) data with the third-order Birch-Murnaghan equation of state (Birch, 1947). The band gaps are obtained using single-point calculations at the optimized lattice constant. A 543 NAO2GTO fitting and cutoff threshold of *ϵ*_Schwarz_ = 10^–5^ are chosen. All screening methods with the default thresholds (*ϵ*_Schwarz_ = *ϵ*_Farfield_ = 10^–6^) are applied.

As summarized in Table 4, our HSE06 results agree satisfactorily with the experimental values. It can also be seen that our results differ slightly from other theoretical values with a difference of 0.01–0.025 Å for *a*_0_, 4–10 GPa for *B*_0_, and 0.02–0.18 eV for *E*_g_, respectively. Actually, such a discrepancy can also be found in other codes (Levchenko et al., 2015; Lin et al., 2020), which can be attributed to the use of different pseudopotentials and basis sets. In the NAO framework, it has been shown that the radial range and shape can influence the final results of DFT calculations (Junquera et al., 2001; Anglada et al., 2002). We use the NAO2GTO fitting to generate new numerical radial functions with a large cutoff radius, which will result in deviations in the HSE06 calculations due to the changes in the radial range and shape of NAOs. As a result, we decide to use 3-6 GTOs with the exponents larger than 0.15 to fit NAOs so that the basis functions do not change significantly. It is important to stress that, we have not observed numerical instability when using truncated CGTOs for HSE06 calculations, but more detailed tests for different systems are still necessary.

**TABLE 4:** Lattice constants *a*_0_ (Å), bulk moduli *B*_0_ (GPa), and band gaps *E*_g_ (eV) for C, Si, SiC, BN, and BP with the cubic diamond structure. Experimental (Expt.) results are taken from in the literature (Heyd and Scuseria, 2004). Theoretical values are from Ref. (Paier et al., 2006a) with plane-wave basis sets, whereas values in parentheses are NAO-based results (Levchenko et al., 2015).

Solid	*a*_0_			*B*_0_			*E*_g_		
	HSE06	Ref.	Expt.	HSE06	Ref.	Expt.	HSE06	Ref.	Expt.
C	3.559	3.549	3.567	457	467	443	5.58	5.49	5.48
Si	5.448	5.435	5.430	101.6	97.7	99.2	1.32	1.14	1.17
		(5.446)			(97.6)			(1.34)	
SiC	4.365	4.348	4.358	222	230	225	2.41	2.39	2.42
BN	3.622	3.603	3.616	391	402	400	6.01	5.98	6.4
BP	4.546	4.521	4.538	163	173	165	2.21	2.16	2.4

### 4.2 Parallel efficiency and computational scaling

In order to illustrate the parallel scalability of our implementation, we perform Γ-only HSE06 calculations for the Si crystal with a supercell containing 512 atoms by using different CPU cores. The default parameters are used for NAO2GTO fitting and integral screening. Our calculations are performed on Intel(R) Xeon(R) CPUs (6258R CPU@2.70GHz). The wall time for building the HFX matrix in the last SCF step is recorded, while the reported wall time for computing the HFX forces only includes the second term in Eq. 13

**FIGURE 3:**
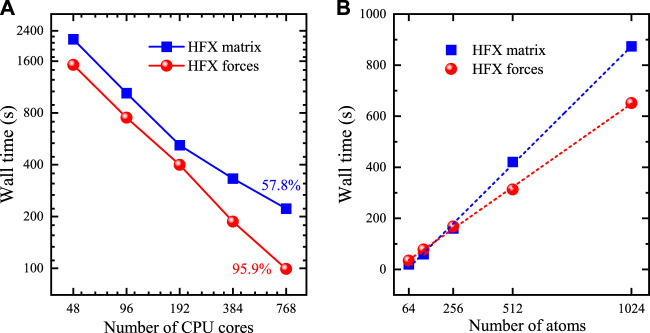
**(A)** The change of wall clock time for HFX matrix and forces with respect to the number of CPU cores for the Si supercell containing 512 atoms. **(B)** The change time of wall clock time for HFX matrix and forces with respect to system size for Si supercells containing from 64 to 1024 atoms running on 240 CPU cores. The dashed lines correspond to a linear fit for the data. All calculations are performed on Intel(R) Xeon(R) CPUs (6258R CPU@2.70GHz).

Figure 3A shows the change of wall times with respect to the number of CPU cores ranging from 48 to 768. The calculation of HFX forces takes 222.1 and 99.2 s for 48 and 768 CPU cores, respectively, which is 1.4–2.2 times faster than the HFX matrix construction (2142.9 and 1521.9 s). In the master-worker dynamic parallelization of HFX force calculation, the load balance can be effectively achieved, and only point-to-point communication of shell quartet indices is needed between the master and worker processes. Thus, the HFX force calculation scales nearly perfectly up to 768 CPU cores with a very high parallel efficiency of 95.9% as expected. However, the parallel efficiency for the construction of HFX matrix is significantly reduced to 57.8%. This reduction can be attributed to all-to-all communications required for building the global HFX matrix, which has been demonstrated in our previous work (Shang et al., 2020).

With such a good parallel scalability, we demonstrate the linear-scaling behavior of our implementation with respect to system size in parallel. We perform a series of Γ-only HSE06 calculations for the Si crystal with different supercells containing from 64 up to 1024 atoms on 240 CPU cores, in which the HFX matrix construction still maintains high parallel efficiency. As shown in Figure 3B, the wall time of both HFX matrix and force computations scale linearly with respect to system size. In particular, the linear-scaling calculation of HFX forces has a smaller prefactor than that of HFX matrix.

### 4.3 Small electron polaron in rutile TiO_2_

As a prototypical photocatalyst, TiO_2_ is one of the most intensively studied materials, and polarons often play a decisive role in its applications (De Lile et al., 2022). For bulk rutile TiO_2_, excess electrons can self-trap to form small polarons associated with local lattice distortion (Setvin et al., 2014). It has shown that hybrid functionals are sufficiently accurate to describe the formation and properties of small polarons in rutile TiO_2_(Janotti et al., 2013; Elmaslmane et al., 2018; De Lile et al., 2022). Herein, we apply our code to investigate the small polaron due to the excess electron in bulk rutile TiO_2_ with HSE06. In all our calculations, the experimental lattice constants of *a* = 4.594 Å and *c* = 2.959 Å are used. The calculated band gap for rutile TiO_2_ is 3.28 eV, slightly higher than the experimental value of 3.03 eV (Amtout and Leonelli, 1995) but lower than the reported HSE06 value of 3.39 eV (Landmann et al., 2012). To simulate the formation of small polaron, a 3 × 3 × 4 supercell containing 216 atoms and −1|*e*| net charge is used for spin-polarized HSE06 calculations. The k-point meshes of 2 × 2 × 2 and 4 × 4 × 4 are chosen for structural relaxation and electronic structure calculations, respectively. One Ti atom is specified an initial displacement ( ∼0.18 Å) for localization of the polaron, and all atomic coordinates are relaxed until the forces acting on each atom are less than 0.04 eV/Å.

**FIGURE 4:**
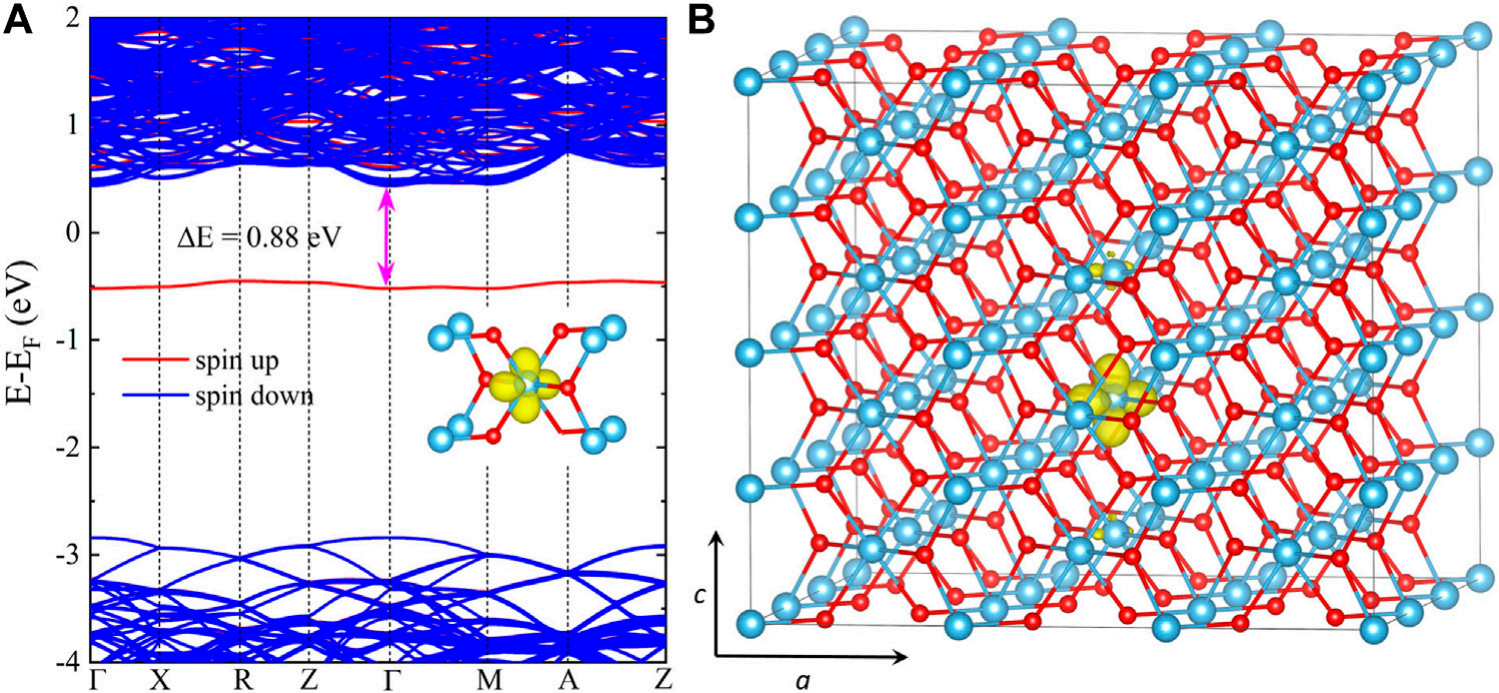
**(A)** HSE06 band structure and **(B)** spin density of the self-trapped electron for the supercell of rutile TiO_2_ with an excess electron. The isosurface is set to be 10% of the maximum charge density, and a 3 × 3 × 4 supercell containing 216 atoms is used.

After full structural optimization, we observe a local lattice distortion around one Ti ion in the electron-doped rutile TiO_2_. Compared to the pristine structure, the two Ti-O bonds perpendicular to the c-axis relax outward, increasing from 1.981 to 1.991 Å in length. In particular, the other four bonds with an initial length of 1.948 Å undergo two distinct changes: two of them increase to 2.011 Å, while the remaining two decrease to 1.891 Å. Figure 4A shows the band structure, where we can find a localized spin-electron state located at roughly 0.88 eV below the conduction band minimum (CBM). We also plot the spin density for this localized state in Figure 4B. As expected, the spin density is localized on the single Ti ion with a local lattice distortion, indicating the formation of a small electron polaron. Our results are in good agreement with the reported HSE06 results, in which an electron polaron state at 0.77 eV below the CBM was predicted by using VASP (Janotti et al., 2013).

## 5 Conclusion

In summary, we have presented an efficient and linear-scaling implementation of analytical gradients of HFX energy for periodic HSE06 calculations within NAOs based on the NAO2GTO scheme. To minimize the errors caused by the NAO2GTO fitting, the original NAOs are replaced by the numerically discretized CGTOs. The ERIs and their derivatives for the HFX term are analytically evaluated with CGTOs, whereas other terms are obtained using discretized CGTOs. Several integral screening methods are utilized to reduce the computational cost of HFX forces, among which the density matrix screening can lead to a linear-scaling calculation of HFX forces with a smaller prefactor compared to the HFX matrix construction. We have demonstrated our implementation can yield accurate results of lattice constants, bulk moduli, and band gaps for several semiconductors. In addition, a master-worker dynamic parallel strategy is employed for computing the HFX forces, which can lead to very high parallel efficiency. We have also studied the small polaronic behavior of excess electrons in rutile TiO_2_, validating the capability of our code for predicting the polarons.

## Data Availability

The original contributions presented in the study are included in the article/Supplementary Material, further inquiries can be directed to the corresponding authors.
